# Impact of community-based interventions on out-of-hospital cardiac arrest outcomes: a systematic review and meta-analysis

**DOI:** 10.1038/s41598-023-35735-y

**Published:** 2023-06-23

**Authors:** Kayla M. Simmons, Sarah M. McIsaac, Robert Ohle

**Affiliations:** 1 Northern Ontario School of Medicine, Sudbury, ON Canada; 2 Department of Critical Care, Department of Anesthesia, Northern Ontario School of Medicine, Sudbury, ON Canada; 3 Department of Emergency Medicine, Northern Ontario School of Medicine, Health Sciences North Research Institute, 56 Walford Road, Sudbury, ON P3E 2H2 Canada

**Keywords:** Epidemiology, Cardiology

## Abstract

Survival following out-of-hospital cardiac arrest (OHCA) remains low, typically less than 10%. Bystander cardiopulmonary resuscitation (CPR) and bystander-AED use have been shown to improve survival by up to fourfold in individual studies. Numerous community-based interventions have been implemented worldwide in an effort to enhance rates of bystander-CPR, bystander-AED use, and improve OHCA survival. This systematic review and meta-analysis aims to evaluate the effect of such interventions on OHCA outcomes. Medline and Embase were systematically searched from inception through July 2021 for studies describing the implementation and effect of one or more community-based interventions targeting OHCA outcomes. Two reviewers screened articles, extracted data, and evaluated study quality using the Newcastle–Ottawa Scale. For each outcome, data were pooled using random-effects meta-analysis. Of the 2481 studies identified, 16 met inclusion criteria. All included studies were observational. They reported a total of 1,081,040 OHCAs across 11 countries. The most common interventions included community-based CPR training (n = 12), community-based AED training (n = 9), and dispatcher-assisted CPR (n = 8). Health system interventions (hospital or paramedical services) were also described in 11 of the included studies. Evidence certainty among all outcomes was low or very low according to GRADE criteria. On meta-analysis, community-based interventions with and without health system interventions were consistently associated with improved OCHA outcomes: rates of bystander-CPR, bystander-AED use, survival, and survival with a favorable neurological outcome. Bystander CPR—14 studies showed a significant increase in post-intervention bystander-CPR rates (n = 285 752; OR 2.26 [1.74, 2.94]; I^2^ = 99%, and bystander AED use (n = 37 882; OR 2.08 [1.44, 3.01]; I^2^ = 54%) and durvival—10 studies, pooling survival to hospital discharge and survival to 30 days (n = 79 206; OR 1.59 [1.20, 2.10]; I^2^ = 95%. The results provide foundational support for the efficacy of community-based interventions in enhancing OHCA outcomes. These findings inform our recommendation that communities, regions, and countries should implement community-based interventions in their pre-hospital strategy for OHCA. Further research is needed to identify which specific intervention types are most effective.

## Introduction

Out-of-hospital cardiac arrest (OHCA) is a leading cause of morbidity and mortality worldwide^1,2^. The incidence of OHCA varies significantly in the literature, affecting 30 to 97 individuals per 100,000 person-years^3^. Survival rates remain low, typically less than 10%^4^. However, these survival rates can vary by up to five-fold in different areas suggesting that there may be opportunities for improvement^2,5,6^. The American Heart Association’s “chain of survival”, was developed in the early 2000s as a systematic and organized community-based approach to help improve the survival rates of OHCA by combining scientific literature and community-based initiatives^7^. Over two decades since the original publication, early bystander cardiopulmonary resuscitation (bystander-CPR) and early defibrillation remain key links in improving OHCA survival^8^. Despite this, the provision of bystander-CPR remains poor. Bystander-CPR is estimated to occur in approximately 40% of OHCAs with some areas reporting rates as low as 19%^1,3^. The rates of bystander automatic external defibrillator (bystander-AED) use are quite variable, ranging from 2 to 37%^3^.

In effort to strengthen the various links in the chain of survival and improve survival following OHCA, communities, regions, and countries across the world have implemented a range of educational interventions. Many studies have reported the efficacy of such interventions in improving rates of bystander-CPR and bystander-AED use as well as important clinical outcomes: overall survival and survival with a favorable neurological outcome following an OHCA^9,10^.

There have been excellent reviews addressing broad community interventions including emergency medical services, fire and police, however there have been few evaluating the efficacy of community-based interventions targeting laypersons^11^. Specifically analysis of initiatives’ characteristics associated with greater impact. This systematic review and meta-analysis explores these factors and the effects of community-based and health system interventions on rates of bystander-CPR, bystander-AED use, survival, and survival with a favorable neurological outcome among individuals experiencing OHCA. These findings will be critical to inform future systemic policy and provide guidance for the implementation of interventions focused on community and national pre-hospital OHCA care to ultimately reduce morbidity and mortality from OHCA.

## Methods

### Search strategy

Electronic searches were conducted in Medline and Embase databases from their earliest record (1946 and 1966 respectively) to December 8, 2021. The search strategy was constructed by one investigator and peer-reviewed using PRESS (Peer Review of Electronic Search Strategies) guidelines by a librarian^12^. We performed citation and conference abstract searches. The full search strategy is presented in eAppendix C in the Online-only Data Supplement.

### Inclusion and exclusion criteria

Included studies needed to report one or more community-based intervention. Community-based interventions were defined as initiatives with a goal of increasing rates of bystander-CPR or bystander-AED use among the lay population. Community included members of a defined geographical community experiencing OHCA. Cities, counties, provinces, states, and countries were all eligible for inclusion as a defined “community”.

To meet inclusion criteria, studies must have reported pre- and post-intervention rates of at least one of the primary outcomes of interest: bystander-CPR and bystander-AED rates. Secondary outcomes of interest for this systematic review were survival to hospital discharge, survival to 30 days, and survival with favourable neurological outcomes (Cerebral Performance Category score of 1 (good cerebral performance) or 2 (moderate cerebral disability))^13^. We excluded studies that only targeted non-lay persons, i.e. dispatcher-assisted CPR, fire fighters, police. Studies exclusively reporting dispatch-assisted CPR or interventions exclusively targeting a population sub group (i.e. first responders, healthcare personnel or school students) did not meet the inclusion criteria for community-based interventions.

### Study selection

Title and abstract screening were completed using a web-based platform Rayyan^14^. Screening was completed independently by two reviewers and blinding was maintained throughout. Conflicts were resolved by discussion until a consensus was reached between investigators.

### Data extraction

Data extraction was completed by two reviewers using a standardized data extraction form. The full data extraction form can be found in eAppendix D. In cases where there were multiple publications describing a single study, the most complete report, in terms of population size and study duration, was used as the primary source.

### Assessment of risk of bias and certainty of evidence

Two reviewers completed risk of bias assessments without blinding for each study using the Newcastle–Ottawa scale, a validated tool for quality assessment of non-randomized studies in meta-analyses^15^. (eAppendix E). Evidence certainty and strength of recommendations for each outcome was evaluated through the GRADE (Grading of Recommendations Assessment, Development and Evaluation) approach by one reviewer and verified by a second reviewer^16^.

### Statistical analysis and data synthesis

Data was synthesized using Review Manager 5.4. Individual and pooled odds ratios (ORs) with 95% confidence intervals (CIs) were calculated for the 4 outcomes of interest using all studies with sufficient data. A random effects meta-analysis was used due to the significant heterogeneity between studies. Heterogeneity was assessed through the I^2^ statistic using criteria from the GRADE handbook: low, ≤ 40%; moderate, 30–60%; substantial, 50–90%; or considerable inconsistency, ≥ 75^17^. Studies were stratified post-hoc into sub-groups by the duration of the study intervention. Publication bias was evaluated by inspection of the funnel plot for each outcome.

This review meets the criteria for the PRISMA (Preferred Reporting Items for Systematic Reviews and Meta-analyses) and MOOSE (Meta-analysis of Observational Studies in Epidemiology) reporting guidelines (eAppendices A & B in the Online-only Data Supplement). The protocol was registered in the PROSPERO database (CRD42021246438). All data are within the manuscript and additional files.

## Results

### Included studies and characteristics

**Figure 1:**
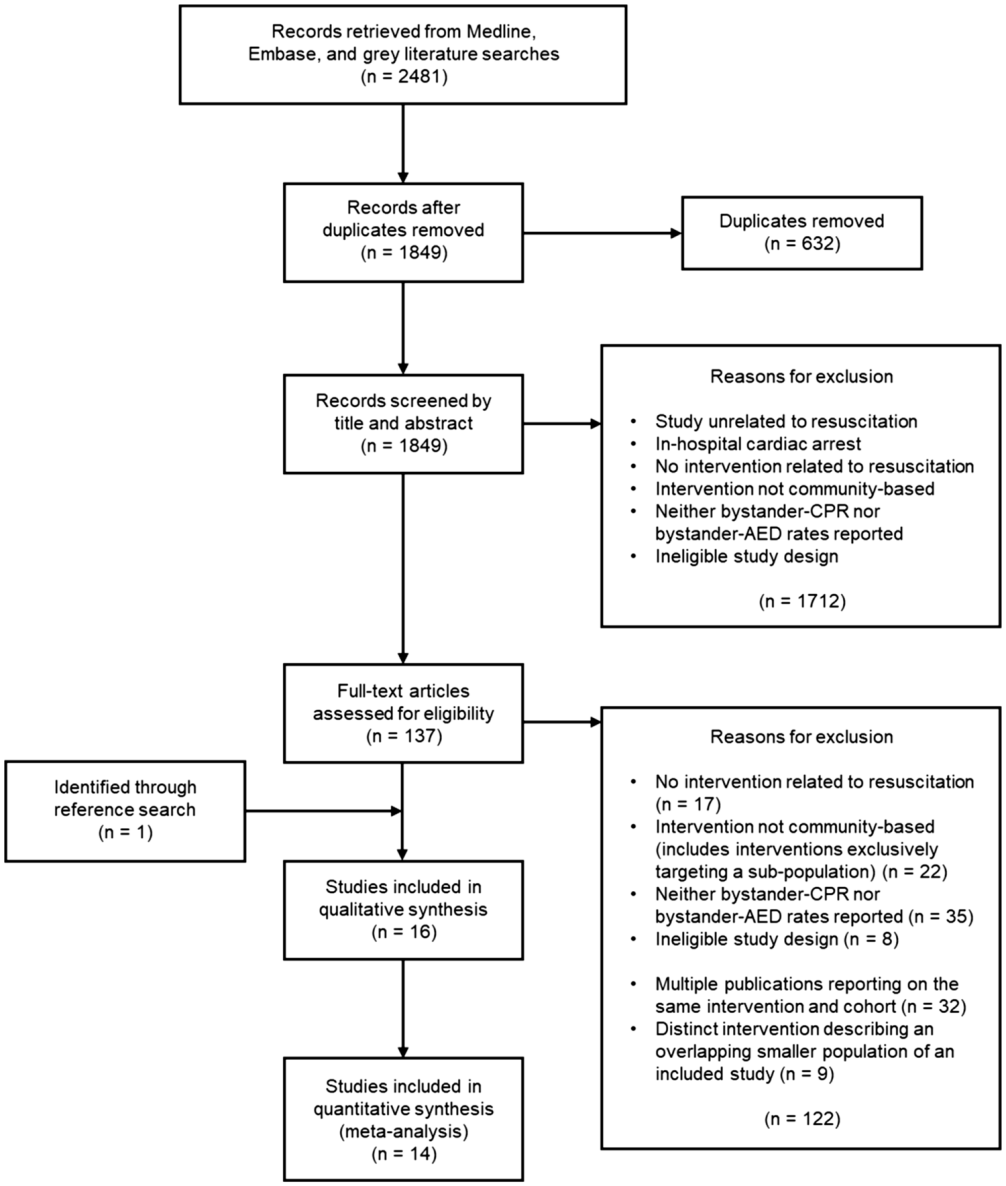
PRISMA flow diagram of study selection.

A total of 2481 records were retrieved. After the removal of 632 duplicates, 1849 articles were screened by title and abstract. Of the 137 articles that underwent full text review(Kappa 0.9), 16 met the pre-determined inclusion criteria(Kappa 0.97) (Fig. 1).

All 16 included studies were observational studies (retrospective n = 9; prospective n = 7). Of these, 6 were conducted in Europe, 6 in the Asia–Pacific region, and 4 in North America. The number of analyzed OHCAs per study ranged from 216 to 816,385 (median = 6103; IQR 3325–38,061) with the total number of included OHCAs reaching 1,091,103. Study and intervention duration ranged from 1.2 to 15 years and 1 day to 15 years, respectively. Full study characteristics are outlined in Table 1.

**Table 1:** Characteristics of included studies.

Study	Location	Study period (years); intervention period* (years)	Study design	Bystander definition^†^	Population	OHCAs in study analysis	Reported outcomes of interest	Additional study outcomes^‡^	NOS score^§^
Franek et al. (2015)^25^	City of Prague, Czech Republic	2003–2013 (11.0)	Retrospective observational	Unspecified	Arrests of cardiac or non-cardiac etiology that received any resuscitation	4529	B-CPR, SFNO	–	6
Wissenberg et al. (2013)^26^	Denmark	2001–2010 (9.6)	Prospective observational	Laypersons only	Non-EMS witnessed arrests of presumed cardiac etiology that received any resuscitation	19,468	B-CPR, B-AED, survival (to 30 days)	Survival (to hospital admission and at 1 year)	6
Dahan et al. (2014)^27^	One city in France	2001–2010 (10.0); 2007–2010 (4.0)	Prospective observational	Unspecified	All OHCA	4176	B-CPR	Survival (to hospital admission)	3
Ristagno et al. (2014)^19^	Cities of Bologna & Bolzano, Italy	2012–2014 (1.2); 2013 (1 week)	Retrospective Observational	Laypersons only	Unspecified	216	B-CPR, survival	–	4
Iwami et al. (2015)^28^	Japan	2005–2012 (8.0)	Prospective observational	Non-EMS and Laypersons	Non-EMS witnessed arrests of presumed cardiac or non-cardiac etiology that received any resuscitation	816,385	B-CPR, SFNO	Prehospital ROSC	8
Lai et al. (2015)^29^	Singapore	2001–2012 (10.7)	Retrospective cohort	Laypersons only	Non-EMS witnessed arrests of presumed cardiac or non-cardiac etiology that received any resuscitation; excluded traumatic arrests	5453	B-CPR, B-AED, survival (to D/C), SFNO	Frequency of EMS and advanced hospital interventions; analysis of factors associated with improved survival	5
Ho et al. (2020)^9^	Singapore	2011–2016 (6.0); 2001–2016 (15.7)^||^	Prospective cohort	Laypersons only	Arrests of presumed cardiac or non-cardiac etiology that received any resuscitation; included those less than 18 years old and traumatic arrests	11,465	B-CPR, B-AED, survival (to D/C), SFNO	Frequency of EMS and advanced hospital interventions	7
Ro et al. (2019)^30^	South Korea	2012–2016 (5.0)	Retrospective observational	Non-EMS and Laypersons	Non-EMS witnessed arrests of presumed cardiac etiology that received any resuscitation	81,250	B-CPR, survival (to D/C), SFNO	–	7
Hollenberg et al. (2008)^10^	Sweden	1992–2005 (14.0)	Retrospective observational	Non-EMS and Laypersons	Arrests of presumed cardiac or non-cardiac etiology for whom an ambulance was called and also received any resuscitation	38 646	B-CPR, survival (to 30 days)	–	7
Mauri et al. (2010)^31^	Southern Switzerland	2005–2009 (5.0); 2006–2009 (3.6)	Prospective observational	Unspecified	Unspecified	1665	B-CPR, survival (to D/C)	–	2
Lin et al. (2016)^32^	4 counties in Taiwan	2012–2015 (4.0)	Prospective observational	Unspecified	All OHCAs that received any resuscitation	37,476	B-CPR, survival (to D/C), SFNO	–	4
Lick et al., (2011)^33^	Anoka County and the City of St. Cloud, Minnesota, United States	2004–2009 (4.6); 2006–2009 (3.0)	Retrospective observational	Laypersons only	Arrests of presumed cardiac etiology that were transported to regional hospitals; excluded those less than 18 years of age	353	B-CPR, survival (to D/C), SFNO	Prehospital ROSC, survival (to admission to intensive care unit), frequency of advanced hospital interventions, financial analysis of hospital interventions	8
Bergamo et al. (2016)^34^	Travis County, Texas, United States	2008–2013 (5.2)	Retrospective observational	Laypersons only	Non-EMS witnessed arrests of presumed cardiac or non-cardiac etiology that received any resuscitation	2474	B-CPR	B-CPR rate change analyzed by high-risk zip codes and initial B-CPR rates	8
van Diepen et al. (2017)^17^	5 States, United States	2011–2015 (5.0)	Prospective observational	Non-EMS and Laypersons	Non-EMS witnessed arrests of non-traumatic presumed cardiac etiology that received any resuscitation	64,988	B-CPR, B-AED, survival (to D/C), SFNO	Frequency of advanced hospital interventions	7
Uber et al. (2018)^5^	Kent County, Michigan, United States	2010–2015 (6.0); 2014 (1 day)	Retrospective observational	Laypersons only	Non-traumatic arrests of presumed cardiac or non-cardiac etiology that received any resuscitation	1486	B-CPR, survival (to D/C), SFNO	Prehospital ROSC	8
Del Rios et al. (2019)^35^	Chicago, Illinois, United States	2013–2016 (3.3)	Retrospective observational	Laypersons only	Non-traumatic arrests of presumed cardiac or non-cardiac etiology that received any resuscitation	6103	B-CPR, B-AED, survival (to D/C), SFNO	Prehospital ROSC; survival (to hospital admission); analysis of factors associated with improved survival	8

*30 days* 30 days following OHCA, *AED* automated external defibrillator, *B-AED* bystander AED use, *B-CPR* bystander CPR, *CBIs* community-based intervention, *CPC* Cerebral Performance Category, *CPR* cardiopulmonary resuscitation, *D/C* discharge from hospital, *EMS* emergency medical services, *HSIs* health system interventions, *NOS* Newcastle–Ottawa Scale, *OHCA* out-of-hospital cardiac arrest, *ROSC* return of spontaneous circulation, *SFNO* survival with favorable neurological outcome (defined as CPC score of 1 or 2).

*Intervention period is specified if it is different than the study period.

^†^Non-EMS includes first responders (i.e., firefighters, police officers) responding to the OHCA; laypersons only excludes first responders.

^‡^Reporting of outcomes among various subgroups (i.e., age, gender, initial cardiac rhythm, Utstein criteria, etc.) have not been included in this list.

^§^Maximum score of 8.

^||^Intervention duration includes the years reported in Lai et al.^29^ as well as the study describes earlier interventions that would contribute to the outcomes noted in Ho et al.^9^.

Interventions reported in each study were categorized as either community-based interventions only (n = 5) or community-based intervention plus health system interventions (n = 11). Health system interventions refer to those that target hospital or paramedicine specific care. Community-based intervention categories included CPR (n = 12) or AED (n = 9) training initiatives, dispatcher-assisted CPR programs (n = 8), school-based training (n = 6), mass media or awareness campaigns (n = 6), public access defibrillation programs (n = 6), brief mass training events (n = 5), legislative changes mandating CPR training for certain sub-populations (n = 3), and notification systems to alert participating bystanders of a nearby OHCA (n = 2). Detailed descriptions of the interventions reported in each study can be found in Table 2.

**Table 2:** Study interventions and outcomes.

Study country	Interventions	Summary of Interventions (no. total duration)	B-CPR Rates (before; after)	B-AED rates (before; after)	Survival rates (before; after)	SFNO (before; after)
Franek et al. (2015) Czech Republic	Community-based 2003: DA-CPR 2003: Public promotion campaign for bystander-CPR (print and social media) Healthcare system 2008: Mechanical CPR devices added to ambulances; enhanced EMS training	2 CBIs & 2 HSIs; 11 years	13%; 82%	–	–	11.6%; 16.8%
Wissenberg et al. (2013) Denmark	Community-based 2001: CPR and AED training programs; 2001–2014 ~ 175 000 CPR certificates annually; 2008–2011 ~ 300 000 CPR certificates annually 2005: Mandatory BLS courses in elementary schools 2006: Mandatory resuscitation course to acquire a driver’s license 2007: PAD program and AED dissemination; registered defibrillators increased from ~ 3000 in 2006 to ~ 15 000 in 2011 2009, 2010: DA-CPR improvements—healthcare professionals at dispatch centres to help callers identify OHCA, AED registry used to guide bystanders to nearby AEDs Healthcare system 2004: Introduction of therapeutic hypothermia in hospitals 2009: Implementation of mobile emergency care units	6 CBIs & 2 HSIs; 9.6 years	20%; 45%	1.0%; 1.9%	To 30 days: 3.5%; 10.8%	–
Dahan et al. (2014) France	Community-based 2007: PAD legislation passed to allow AED use by laypersons 2007: Public information campaign regarding the chain of survival	2 CBIs; 4 years	23%; 31%	–		–
Ristagno et al. (2014) Italy	Community-based 2013: “Viva!”, weeklong awareness and mass resuscitation training initiative	2 CBIs; 1 week	18%; 27%*	–	Survival unchanged*^†^	–
Iwami et al. (2015) Japan	Community-based 2004: PAD legislation passed; ~ 350 000 AEDs deployed 2005: CPR and AED training programs in community and schools; total of ~ 4 million trained/year; 1-h, 3-h, and 8-h options 2005: DA-CPR with traditional CPR instructions 2006: DA-CPR with COCPR instructions	5 CBIs; 8 years	35%; 47%	–	–	1.0%; 1.9%
Lai et al. (2015) Singapore	Community-based 2001: Ongoing expansion of CPR and AED training centres and programs; more than 30 000 trained annually 2006: Widespread distribution of AEDs in public locations (airports, sports facilities, hotels, malls, etc.) 2011: Introduction of annual awareness campaign, National Life Saving Day Healthcare system 2001: Motorcycle Fast Response Paramedics (FRPs) introduced over several years 2004: Paramedics certified to give IV epinephrine and use laryngeal mask airways 2007: Introduction of therapeutic hypothermia, ECMO, and PCI 2011: Mechanical CPR devices added to ambulances	4 CBIs & 4 HSIs; 10.7 years	20%; 22%	0.0%; 1.0%	To D/C: 1.6%; 3.2%	1.2%; 1.8%
Ho et al. (2020) Singapore	Community-based* 2011: Introduction of annual awareness campaign, National Life Saving Day 2012: DA-CPR with COCPR instructions 2014: Dispatcher-Assisted first Responder (DARE) training initiative—CPR and AED training free to public and mandated for school children; ~ 50 000 trained from 2014–2016 2015: PAD program (Save-a-Life initiative) and notification system– over 360 AEDs were placed and registered in residential areas, myResponder mobile application to alert participating laypersons within 400 m of an OHCA and the location of the closest AED; participating taxi drivers equipped with AEDs within a 1.5 km radius are also alerted Healthcare system* 2011: Mechanical CPR devices added to ambulances 2012: Fire Bikers Scheme implemented where fire and rescue first responders were dispatched ahead of the ambulance in times of heavy traffic 2014: Intraosseous access was permitted in ambulances *Please also see interventions listed under Lai et al., 2015	7 CBIs & 3 HSIs; 15.7 years	22%; 56%	1.7%; 4.6%	To D/C: 3.5%; 6.5%	1.8%; 4.4%
Ro et al. (2019) South Korea	Community-based 2012: DA-CPR implemented nationwide by the National Fire Agency 2013: CPR and AED training programs; annual layperson training numbers increased from ~ 300 000 in 2013 to ~ 600 000in 2016 (~ 2 000 000 over 4 years) Healthcare system 2013: Mandated CPR and AED training for first responders (i.e., firefighters, police, lifeguards, etc.)	3 CBIs & 1 HSIs; 5 years	35%; 65%	–	To D/C: 10.0%; 11.1%*	5.4%; 7.1%
Hollenberg et al. (2008) Sweden	Community-based 1992: CPR training program; utilized a train-the-trainer approach; shortened from 3 h to 35 min in 2006; ~ 2 000 000 trained; AED content added in 1996 1997: School based CPR and AED training programs initiated 1997: DA-CPR Healthcare system ALS training for critical care nursing staff Higher rates of post-resuscitation interventions (hypothermia, revascularization)	4 CBIs & 2 HSIs; 14 years	31%; 50%	–	To 30 days: 4.8%; 7.3%	–
Mauri et al. (2010) Switzerland	Community-based 2006: Early Resuscitation and Defibrillation Program—CPR and AED training, layperson notification system 2006: Compulsory CPR training in school youth	4 CBIs; 3.6 years	11%; 18%	–	To D/C: 2.0%; 6.1%	–
Lin et al. (2016) Taiwan	Community-based 2012: COCPR training for laypersons 2013: PAD and Good Samaritan legislation passed 2013: DA-CPR	3 CBIs; 4 years	25%; 33%	–	To D/C: 5.7%; 7.6%	1.7%; 3.2%
Lick et al. (2011) United States	Take Heart America (THA) program A community-wide, systems-based approach based on the 2005 American Heart Association (AHA) CPR and Emergency Cardiovascular Care guidelines Community-based 2006: Initiation of several CPR and AED training initiatives (laypersons, high school students and their families, family members of patients who recently experienced an OHCA); 28 041 trained from September 2006 to December 2008 2006: AED distribution in high-traffic public buildings (132 AEDs deployed) 2006: More than 60 media publications on OHCA (print, radio, television) 2008: “CPR Goes to College” pilot program at local university; ~ 10 000 trained Healthcare system AEDs placed in all first-responder vehicles (fire, police); first-responders trained in high performance CPR Introduction of therapeutic hypothermia and enhanced rates of advanced hospital interventions at specialized cardiac arrest centers (CACs)	5 CBIs & 3 HSIs; 3 years	20%; 29%	–	To D/C: 8.5%; 19.4%	SFNO unchanged*^,†^
Bergamo et al. (2016) United States	Community-based 2008: Launch of the TAKE10 Program (under the Take Heart America organization) – CPR training program using a train-the-trainer approach to deliver 10-min sessions to learn COCPR (~ 2000 trained annually) 2011: Awareness campaign endorsed by community officials	2 CBIs; 5.2 years	42%; 47%	–	–	–
van Diepen et al. (2017) United States	HeartRescue Project 2010: Various community-based and healthcare interventions started at different times across the 5 participating States (Arizona, Minnesota, North Carolina, Pennsylvania, and Washington) Community-based For example, CPR and AED training, PAD programs, DA-CPR, mass events, and awareness campaigns Healthcare system For example, enhanced EMS training, collaboration, and coordination among resuscitation centres	6 CBIs & 2 HSIs; 5 years	42%; 43%	3.2%; 5.6%	To D/C: 13.7%; 10.5%	10.4%; 8.9%
Uber et al. (2018) United States	Community-based May 21, 2014: One-day mass COCPR training event at 7 public locations; trained a convenience sample of 2253 individuals	1 CBI; 1 day	37%; 36%*	–	To D/C: 9.9%; 10.1%*	8.3%; 8.7%*
Del Rios et al. (2019) United States	Community-based 2013: Illinois Heart Rescue Program (ILHR) – CPR training programs initiated, specifically targeting communities with high OHCA incidence and low bystander-CPR rates (~ 9000 trained annually); public awareness campaign 2014: DA-CPR training and protocol update (“no, no, go” method); DA-CPR was initially implemented prior to the study period Healthcare system 2013: Updated hospital designations to ensure patient transport to the appropriate facility 2015: Updated ROSC protocol in the hospital	3 CBIs & 2 HSIs; 3.3 years	11%; 19%	0.9%; 1.3%*	To D/C: 7.3%; 9.9%*	4.3%; 6.4%

*30 days* 30 days following OHCA, *AED* automated external defibrillator, *ALS* advanced life support, *B-AED* bystander AED use, *B-CPR* bystander CPR, *BLS* basic life support; *CBIs* community-based intervention, *COCPR* compression only CPR, *CPC* Cerebral Performance Category, *CPR* cardiopulmonary resuscitation, *D/C* discharge from hospital, *DA-CPR* dispatcher-assisted CPR, *EMS* emergency medical services, *HSIs* health system interventions, *OHCA* out-of-hospital cardiac arrest, *PAD* public access defibrillation, *ROSC* return of spontaneous circulation.

*Rate change *not* statistically significant.

^†^Study reports that rates were not significantly changed. No exact rates provided for before or after periods.

### Outcomes

**Figure 2:**
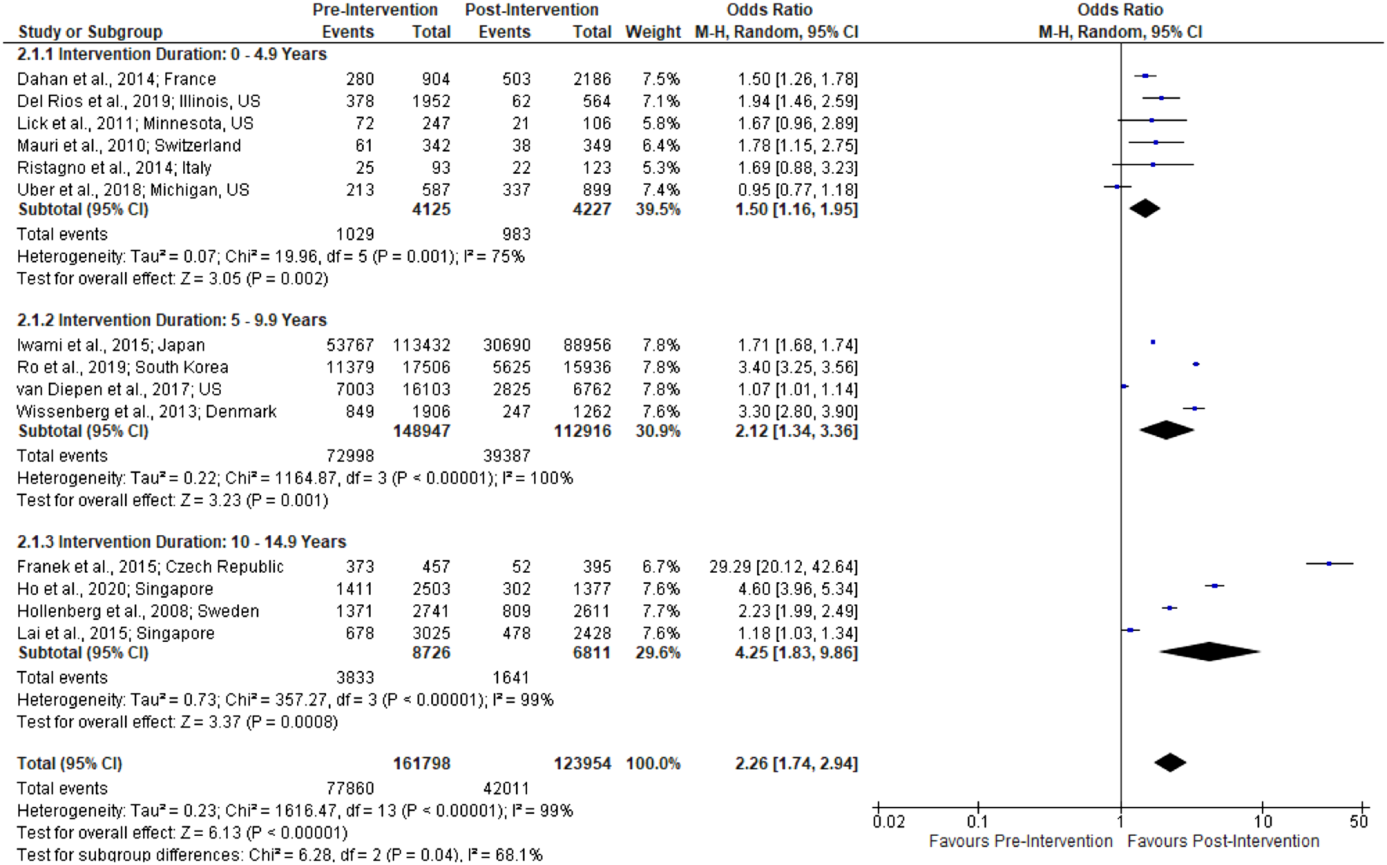
Forest plot of studies reporting bystander-CPR rates stratified by intervention duration.

**Figure 3:**
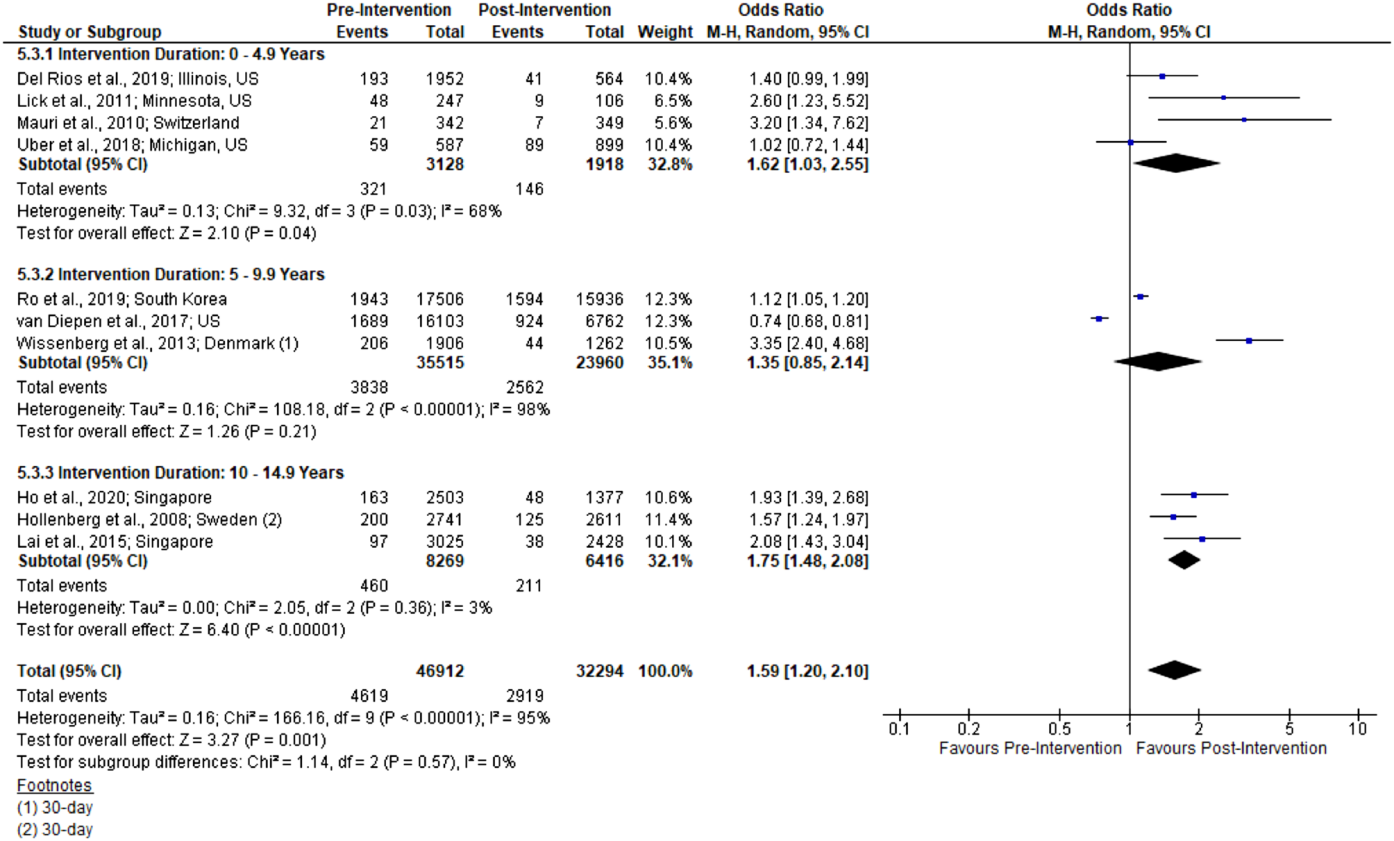
Forest plot of studies reporting survival rates stratified by intervention duration.

**Figure 4:**
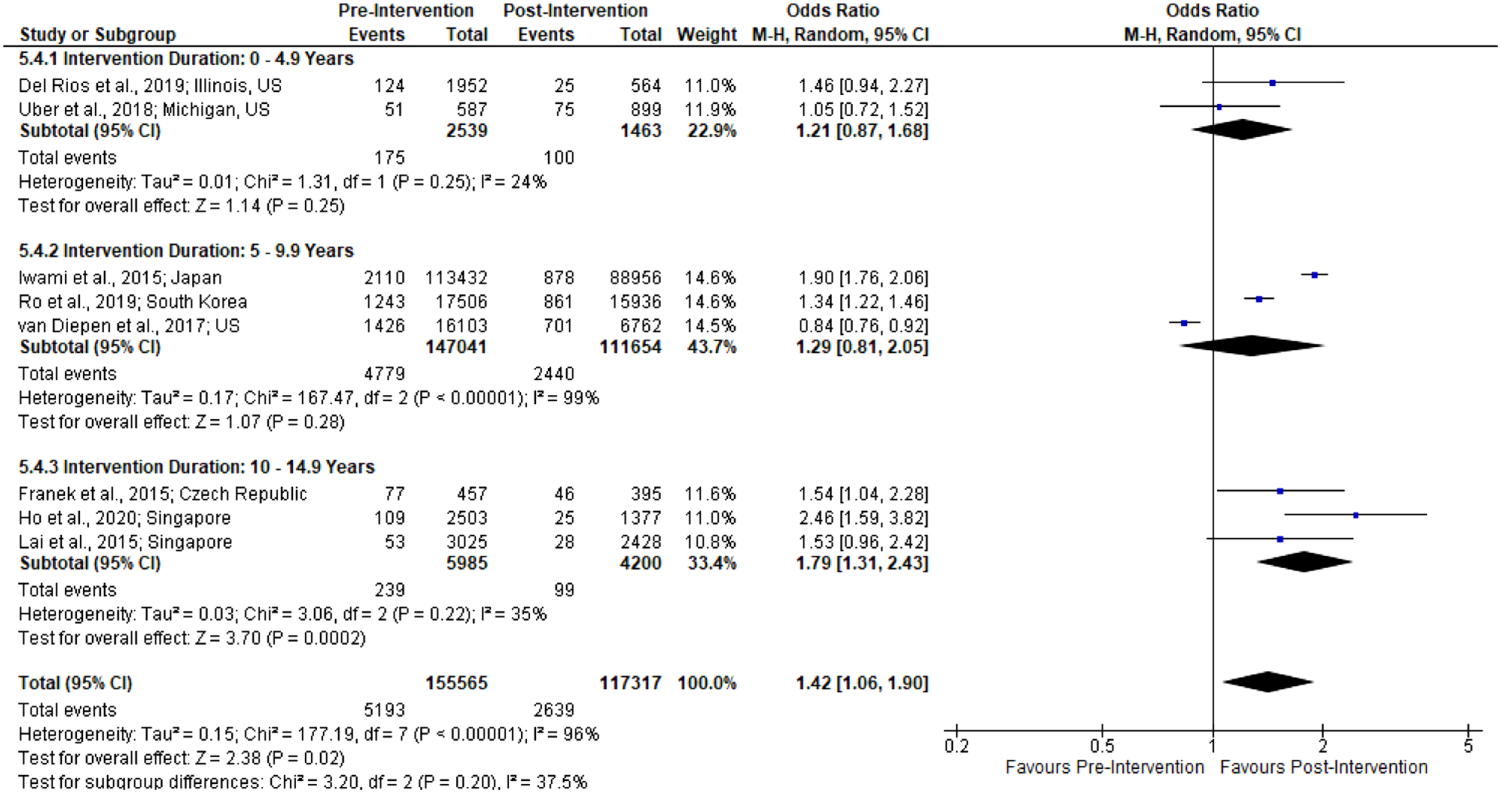
Forest plot of studies reporting survival with favorable neurological outcome rates stratified by intervention duration.

Table 1 outlines the outcomes reported in each study and Table 2 shows the corresponding pre- and post-intervention rates. Results of meta-analyses are presented in forest plots (Figs. 2, 3, 4) and summarized in Table 3.

**Table 3:** GRADE summary of findings.

*Patient or population* adults and children with cardiac arrest in the out-of-hospital setting *Intervention* community-based interventions (CBIs) with or without health system interventions (HSIs) *Comparison* prior to or without CBIs or HSIs					
Outcomes	№ of participants (studies) Follow up	Certainty of the evidence (GRADE)	Relative effect (95% CI)	Anticipated absolute effects	
				Risk prior to CBIs or HSIs	Risk difference after CBIs or HSIs
Bystander-CPR rates (critical outcome)	285,752 (14 observational studies)	⨁⨁◯◯ LOW*^,^^†,‡^^,^^§^	OR 2.26 (1.74 to 2.94)	339 per 1000	198 more per 1000 (133 to 262 more)
Survival rates (critical outcome)	79,206 (10 observational studies)	⨁◯◯◯ VERY LOW*^,^^†,§^	OR 1.59 (1.20 to 2.10)	90 per 1000	46 more per 1000 (16 to 82 more)
Survival with favorable neurological outcome rates (critical outcome)	272,882 (8 observational studies)	⨁◯◯◯ VERY LOW*^,^^†,§^	OR 1.42 (1.06 to 1.90)	22 per 1000	9 more per 1000 (1 to 19 more)
Bystander-AED rates (important outcome)	37,882 (5 observational studies)	⨁◯◯◯ VERY LOW*	OR 2.08 (1.44 to 3.01)	21 per 1000	22 more per 1000 (9 to 39 more)

*The risk in the intervention group* (and its 95% confidence interval) is based on the assumed risk in the comparison group and the *relative effect* of the intervention (and its 95% CI).

*CI* confidence interval, *OR* odds ratio.

GRADE Working Group grades of evidence.

*High certainty* We are very confident that the true effect lies close to that of the estimate of the effect.

*Moderate certainty* We are moderately confident in the effect estimate: The true effect is likely to be close to the estimate of the effect, but there is a possibility that it is substantially different.

*Low certainty* Our confidence in the effect estimate is limited: The true effect may be substantially different from the estimate of the effect.

*Very low certainty* We have very little confidence in the effect estimate: The true effect is likely to be substantially different from the estimate of effect.

*Serious risk of bias: failure to adequately control or adjust for confounding factors due to study design.

^†^Serious inconsistency: considerable heterogeneity present (defined as I-squared 75–100%); some but not all heterogeneity explained with sub-group analysis.

^‡^Upgraded for strong association.

^§^Upgraded for dose–response relationship.

#### Bystander-CPR (critical outcome)

Bystander-CPR rates were reported in all 16 studies of which 14 of these studies showed significant improvement in bystander-CPR (p < 0.05). Meta-analysis of 14 studies showed a significant difference in post-intervention bystander-CPR rates (n = 285 752; OR 2.26 [1.74, 2.94]; I^2^ = 99%; Fig. 2). All three intervention duration subgroups were significantly improved following interventions with increasing effect size noted with greater intervention duration: 0–4.9 years (OR 1.50 [1.16, 1.95]), 5–9.9 years (OR 2.12 [1.34, 3.36]), and 10–15 years (OR 4.25 [1.83, 9.86]).

#### Bystander AED Rates (important outcome)

Five of the included studies reported data on bystander-AED rates. All 5 reported increases; 4 were statistically significant while the last was not. Meta-analysis of the pooled results showed a significant change in the post-intervention period (n = 37 882; OR 2.08 [1.44, 3.01]; I^2^ = 54%).

#### Survival (critical outcome)

Survival was reported in 12 studies. Two studies reported survival to 30 days; both showed significant increases (p < 0.05). The remaining 10 studies described survival to hospital discharge. Among these, 5 reported significant increases and 4 reported non-significant increases. The final study had a significant decrease in survival to discharge (13.7% to 10.5%; p < 0.01) though the findings did not remain significant following multivariable adjustment (p = 0.08)^17^. Meta-analysis included 10 studies, pooling survival to hospital discharge and survival to 30 days (n = 79 206; OR 1.59 [1.20, 2.10]; I^2^ = 95%; Fig. 4). Among the sub-groups, 0–4.9 years (1.62 [1.03, 2.55]) and 10–15 years (OR 1.75 [1.48, 2.08]) were significantly improved as compared to 5–9.9 years (1.35 [0.85, 2.14]).

#### Survival with favorable neurological outcome (critical outcome)

Survival with favorable neurological outcome, CPC 1 or 2, was reported in 9 studies. Of these, 7 had significant improvement (p < 0.05). One study noted a negligible increase (8.3% to 8.7%; p = 0.87)^5^. The last study noted a significant decrease (10.4% to 8.9%; p < 0.01) though the decrease was not statistically significant after their multivariable adjustment (p = 0.07)^17^. On meta-analysis of 8 studies, the pooled results were significant (n = 272,882; OR 1.42 [1.06, 1.90]; I^2^ = 96%). Among the sub-groups only 10–15 years was significant (OR 1.79 [1.31, 2.43]); the effect size increased with intervention duration.

### Risk of bias and certainty of evidence

The Newcastle–Ottawa tool was used to assess the quality of included studies on a scale of 0 to 8. Nine studies received scores of 7 or 8; three a score of 5 or 6, and the remaining four a score of 3 or 4 (Table 4).

**Table 4:** Summary of risk of bias assessment for observational studies using the Newcastle–Ottawa Scale (NOS).

Study ID	Selection (max 4 stars)	Comparability (max 1 star)	Outcome (max 3 stars)	Total Score (max 8)
Franek et al. (2015)	★★★★		★★	6
Wissenberg et al. (2013)	★★★★		★★	6
Dahan et al. (2014)	★		★★	3
Ristagno et al. (2014)	★★★		★	4
Iwami et al. (2015)	★★★★	★	★★★	8
Lai et al. (2015)	★★★		★★	5
Ho et al. (2020)	★★★★	★	★★	7
Ro et al. (2019)	★★★★	★	★★	7
Hollenberg et al. (2008)	★★★★	★	★★	7
Mauri et al. (2010)	★		★★	3
Lin et al. (2016)	★★		★★	4
Lick et al. (2011)	★★★★	★	★★★	8
Bergamo et al. (2016)	★★★★	★	★★★	8
van Diepen et al. (2017)	★★★★	★	★★	7
Uber et al. (2018)	★★★★	★	★★★	8
Del Rios et al. (2019)	★★★★	★	★★★	8

The certainty of effect size for each outcome was evaluated according to GRADE criteria and was found to be low or very low certainty in all outcomes of interest (Table S1 in the Online-only Data Supplement). The main shortcomings across outcomes were the risk of bias and inconsistency due to significant heterogeneity among studies. A dose–response gradient was noted for rates of bystander-CPR, survival, and survival with favorable neurological outcome.

### Heterogeneity and sensitivity analysis

Heterogeneity for pooled bystander-AED analysis was moderate (I^2^ = 54%). Pooled I^2^ values for the other 3 outcomes were considerable: bystander-CPR (I^2^ = 99%), survival (I^2^ = 95%), and survival with favorable neurological outcome (I^2^ = 96%). Stratification by intervention duration accounted for some heterogeneity. Among the 9 sub-groups heterogeneity decreased to low in 3 groups (survival 10–15 years; survival with favorable neurological outcome 0–4.9 and 10–15 years) and to substantial in 2 groups (bystander-CPR 0–4.9 years; survival 0–4.9 years). Despite the heterogeneity, the authors chose to present the results of the meta-analyses according to the following rationale. First, there is a clear positive trend among all outcomes. Second, there is a pattern among the stratified outcomes (larger effect size with greater intervention duration) suggesting the outcomes are not random; further, this dose–response gradient may be critical to informing the duration of future initiatives. Third, the observed heterogeneity is accounted for when judging the certainty of evidence through the GRADE approach and contributes to our low certainty in the effect size of outcomes. As such, the meta-analyses are presented in this paper, but the pooled estimates must be interpreted with caution^18^.

## Discussion

### Principle findings

This systematic review and meta-analysis included 16 studies reporting on 1,091,103 OHCAs in 11 countries. The findings suggest that community-based interventions and community-based interventions plus health system interventions are associated with significant improvement in all 4 outcomes of interest. The data suggest a relationship between intervention duration and effect size as noted in meta-analyses and examining commonalities among the least effective studies^5,19^. Further, community-based interventions plus health system interventions were noted to have a slightly larger effect suggesting the importance of bundling interventions.

### Findings in the context of previous work

Our review is the first to evaluate the efficacy of community-based interventions, defined as those explicitly targeting laypersons. There have been two systematic reviews (one with meta-analysis) assessing the effects of broader system-based interventions including those targeting first-responders, EMS, and hospital systems; many of the included studies in both reviews lacked interventions targeting laypersons. However, these reviews reported similar improvements in system-level and clinical outcomes as those described in our review: improved bystander-CPR, survival, and survival with favorable neurological outcome^20,21^. Thus, our findings provide further support for the efficacy of health system interventions and foundational evidence for the efficacy of community-based interventions.

We observed a pattern of greater improvement among studies which bundled the 3 most common community-based interventions: CPR training, AED training, and dispatcher-assisted CPR (included in 12, 9, and 8 studies respectively). A recent systematic review compared outcomes between EMS systems with dispatch-assisted CPR to EMS systems where it is not offered^22^. They found that dispatcher-assisted CPR is associated with significantly higher bystander-CPR rates (n = 9 studies; OR 3.10 [2.25, 4.25]) but not increased rates of high-quality bystander-CPR; nor was there any significant improvement in survival following OHCA^22^. It is possible that this bundle of 3 interventions has a synergistic effect; prior resuscitation training provides competency and higher quality CPR while dispatcher-assisted CPR can prompt action resulting in a greater overall intervention effect.

We found a temporal trend between intervention duration and enhanced outcomes. There are several possible explanations for this trend. A lack of training is a significant barrier to providing bystander-CPR and bystander-AED^23,24^. Cultural relevance of curriculum, costs, and language concerns have been shown to impact CPR/AED training attainment. With long-term training initiatives, a greater proportion of the population could be trained, increasing the likelihood that a qualified bystander will be present at an OHCA event. It is also possible that with prolonged exposure to mass media and awareness campaigns, there is a change in the culture regarding resuscitation that increases the comfort of bystanders to perform CPR^23,24^.

### Implications

The GRADE Evidence to Decision framework was utilized to inform recommendations given our findings in the context of broader considerations (full description in eAppendix F of the Online-only Data Supplement). The observational design and the high heterogeneity between included studies were the primary contributors that reduced the evidence certainty of outcomes to low or very low.

We strongly recommend based on low quality evidence that communities, regions, and countries assess their current pre-hospital strategy for OHCA care and consider implementing community-based interventions, especially when combined with health system interventions, to improve OHCA outcomes.

### Strengths and limitations

The strengths of this review include robust methods to select, appraise, and analyze study findings. Included studies provide generalizable results as they span 24 years, 11 countries, include over one million OHCAs, and describe a range of community-based interventions and health system interventions. As with all research, the findings of this review should be considered in the context of study limitations. The observational design of studies resulted in a high risk of bias and the potential for confounding variables. Temporal trends in CPR and OHCA outcomes may limit the certainty of the effects of the interventions in the included studies. Significant heterogeneity between studies also reduces the certainty of evidence among outcomes of interest. There is substantial heterogeneity in the patient populations (non-EMS witnessed arrests, arrests that received resuscitation, all OHCA, OHCA transported to hospital) which may bias the results of this meta-analysis. There exists potential for misclassification of “laypersons”—international EMS personnel and reporting systems may have differing definitions.

### Future direction

Implementation of community-based interventions for OHCA is needed for high-risk communities, particularly those with suboptimal EMS services. However, additional data on cost-effectiveness (costs per life saved) is needed. Moreover, with advancements in technology, novel interventions to engage the community in resuscitation need to be investigated, including crowdsourcing technologies, drone-delivered AEDs, AED registries and pocket AEDs.

## Conclusion

Community-based interventions are associated with significant improvement in all 4 outcomes of interest: rates of bystander-CPR, bystander-AED use, survival, and survival with a favorable neurological outcome following OHCA. We recommend that communities and policymakers assess their current pre-hospital strategy for OHCA care and consider implementing a bundle of community-based interventions. For greatest impact, initiatives should be sustainable and continued long term.

## Supplementary Information

Supplementary Information.

## Data Availability

All data are within the manuscript and additional files.
