# Supplementary material for: Impact of community-based interventions on out-of-hospital cardiac arrest outcomes: a systematic review and meta-analysis
Source: Sci Rep. 2023 Jun 23;13:10231. doi: 10.1038/s41598-023-35735-y (PMC10290111; doi:10.1038/s41598-023-35735-y)
Supplement: Supplementary file 1 — Supplementary Information. [file 41598_2023_35735_MOESM1_ESM.docx]

**SUPPLEMENTAL MATERIAL**

[eAppendix A: Preferred Reporting Items for Systematic Reviews and Meta-analyses (PRISMA) Checklist 2](#_Toc92473689)

[eAppendix B: Meta-analysis of Observational Studies in Epidemiology (MOOSE) Checklist 6](#_Toc92473690)

[eAppendix C: Full Search Strategy 8](#_Toc92473691)

[eAppendix D: Data Extraction Form 11](#_Toc92473692)

[eAppendix E: Quality Assessment Criteria 15](#_Toc92473693)

[eAppendix F: GRADE Evidence to Decision Framework 17](#_Toc92473694)

[Supplemental Table 1. Evidence Profiles for Outcomes. 24](#_Toc92473695)

[Supplemental Figure 1. Forest plot of studies reporting Bystander-AED rates 26](#_Toc92473698)

eAppendix A: Preferred Reporting Items for Systematic Reviews and Meta-analyses (PRISMA) Checklist

| Section and Topic | Item # | Checklist item | Reported location / page(s) |
| --- | --- | --- | --- |
| TITLE | | |  |
| Title | 1 | Identify the report as a systematic review. | 1 |
| ABSTRACT | | |  |
| Abstract | 2 | See the PRISMA 2020 for Abstracts checklist. |  |
| INTRODUCTION | | |  |
| Rationale | 3 | Describe the rationale for the review in the context of existing knowledge. | 2 |
| Objectives | 4 | Provide an explicit statement of the objective(s) or question(s) the review addresses. | 2 |
| METHODS | | |  |
| Eligibility criteria | 5 | Specify the inclusion and exclusion criteria for the review and how studies were grouped for the syntheses. | 5-7 |
| Information sources | 6 | Specify all databases, registers, websites, organisations, reference lists and other sources searched or consulted to identify studies. Specify the date when each source was last searched or consulted. | 4 |
| Search strategy | 7 | Present the full search strategies for all databases, registers and websites, including any filters and limits used. | eAppendix C |
| Selection process | 8 | Specify the methods used to decide whether a study met the inclusion criteria of the review, including how many reviewers screened each record and each report retrieved, whether they worked independently, and if applicable, details of automation tools used in the process. | 5-7 |
| Data collection process | 9 | Specify the methods used to collect data from reports, including how many reviewers collected data from each report, whether they worked independently, any processes for obtaining or confirming data from study investigators, and if applicable, details of automation tools used in the process. | 6-7 |
| Data items | 10a | List and define all outcomes for which data were sought. Specify whether all results that were compatible with each outcome domain in each study were sought (e.g. for all measures, time points, analyses), and if not, the methods used to decide which results to collect. | 5-6, Table 1 |
|  | 10b | List and define all other variables for which data were sought (e.g. participant and intervention characteristics, funding sources). Describe any assumptions made about any missing or unclear information. | 5-6;  eAppendix D |
| Study risk of bias assessment | 11 | Specify the methods used to assess risk of bias in the included studies, including details of the tool(s) used, how many reviewers assessed each study and whether they worked independently, and if applicable, details of automation tools used in the process. | 7;  eAppendix E |
| Effect measures | 12 | Specify for each outcome the effect measure(s) (e.g. risk ratio, mean difference) used in the synthesis or presentation of results. | 7 |
| Synthesis methods | 13a | Describe the processes used to decide which studies were eligible for each synthesis (e.g. tabulating the study intervention characteristics and comparing against the planned groups for each synthesis (item #5)). | 7 |
|  | 13b | Describe any methods required to prepare the data for presentation or synthesis, such as handling of missing summary statistics, or data conversions. | 6-7 |
|  | 13c | Describe any methods used to tabulate or visually display results of individual studies and syntheses. | 6-7 |
|  | 13d | Describe any methods used to synthesize results and provide a rationale for the choice(s). If meta-analysis was performed, describe the model(s), method(s) to identify the presence and extent of statistical heterogeneity, and software package(s) used. | 6-7 |
|  | 13e | Describe any methods used to explore possible causes of heterogeneity among study results (e.g. subgroup analysis, meta-regression). | 6-7, 11-12 |
|  | 13f | Describe any sensitivity analyses conducted to assess robustness of the synthesized results. | 7, 11-12 |
| Reporting bias assessment | 14 | Describe any methods used to assess risk of bias due to missing results in a synthesis (arising from reporting biases). | 7 |
| Certainty assessment | 15 | Describe any methods used to assess certainty (or confidence) in the body of evidence for an outcome. | 10-11, 14; Table S1 |
| RESULTS | | |  |
| Study selection | 16a | Describe the results of the search and selection process, from the number of records identified in the search to the number of studies included in the review, ideally using a flow diagram. | 8, Figure 1 |
|  | 16b | Cite studies that might appear to meet the inclusion criteria, but which were excluded, and explain why they were excluded. | 10 |
| Study characteristics | 17 | Cite each included study and present its characteristics. | 8, Table 1 |
| Risk of bias in studies | 18 | Present assessments of risk of bias for each included study. | 10-11, Table 4 |
| Results of individual studies | 19 | For all outcomes, present, for each study: (a) summary statistics for each group (where appropriate) and (b) an effect estimate and its precision (e.g. confidence/credible interval), ideally using structured tables or plots. | Table 2, Figures 2-4; Figure S1 |
| Results of syntheses | 20a | For each synthesis, briefly summarise the characteristics and risk of bias among contributing studies. | Table S1 |
|  | 20b | Present results of all statistical syntheses conducted. If meta-analysis was done, present for each the summary estimate and its precision (e.g. confidence/credible interval) and measures of statistical heterogeneity. If comparing groups, describe the direction of the effect. | Figures 2-4; Figure S1, Table S1 |
|  | 20c | Present results of all investigations of possible causes of heterogeneity among study results. | 11-12 |
|  | 20d | Present results of all sensitivity analyses conducted to assess the robustness of the synthesized results. | 11-12 |
| Reporting biases | 21 | Present assessments of risk of bias due to missing results (arising from reporting biases) for each synthesis assessed. | Table S1 |
| Certainty of evidence | 22 | Present assessments of certainty (or confidence) in the body of evidence for each outcome assessed. | Table S1 |
| DISCUSSION | | |  |
| Discussion | 23a | Provide a general interpretation of the results in the context of other evidence. | 11-14 |
|  | 23b | Discuss any limitations of the evidence included in the review. | 14-15 |
|  | 23c | Discuss any limitations of the review processes used. | 15 |
|  | 23d | Discuss implications of the results for practice, policy, and future research. | 14-15 |
| OTHER INFORMATION | | |  |
| Registration and protocol | 24a | Provide registration information for the review, including register name and registration number, or state that the review was not registered. | 3 |
|  | 24b | Indicate where the review protocol can be accessed, or state that a protocol was not prepared. | 3 |
|  | 24c | Describe and explain any amendments to information provided at registration or in the protocol. | 3 |
| Support | 25 | Describe sources of financial or non-financial support for the review, and the role of the funders or sponsors in the review. | 16 |
| Competing interests | 26 | Declare any competing interests of review authors. | 16 |
| Availability of data, code and other materials | 27 | Report which of the following are publicly available and where they can be found: template data collection forms; data extracted from included studies; data used for all analyses; analytic code; any other materials used in the review. | 3 |

eAppendix B: Meta-analysis of Observational Studies in Epidemiology (MOOSE) Checklist

| **Item No** | **Recommendation** | **Reported on Page No** |
| --- | --- | --- |
| **Reporting of Background** | | |
| 1 | Problem definition | 3 |
| 2 | Hypothesis statement | 3 |
| 3 | Description of study outcome(s) | 5 |
| 4 | Type of exposure or intervention used | 5 |
| 5 | Type of study designs used | 5-6 |
| 6 | Study population | 5 |
| **Reporting of Search strategy** | | |
| 7 | Qualifications of searchers (eg, librarians and investigators) | 4 |
| 8 | Search strategy, including time period included in the synthesis and key words | 4, eAppendix C |
| 9 | Effort to include all available studies, including contact with authors | 4, 7 |
| 10 | Databases and registries searched | 4, eAppendix C |
| 11 | Search software used, name and version, including special features used (eg, explosion) | 4, 6, eAppendix C |
| 12 | Use of hand searching (eg, reference lists of obtained articles) | 4, Figure 1 |
| 13 | List of citations located and those excluded, including justification | Figure 1, eAppendix C |
| 14 | Method of addressing articles published in languages other than English | 5-6 |
| 15 | Method of handling abstracts and unpublished studies | 5-6 |
| 16 | Description of any contact with authors | 7 |
| **Reporting of Methods** | | |
| 17 | Description of relevance or appropriateness of studies assembled for assessing the hypothesis to be tested | 5-7 |
| 18 | Rationale for the selection and coding of data (eg, sound clinical principles or convenience) | 6-7 |
| 19 | Documentation of how data were classified and coded (eg, multiple raters, blinding and interrater reliability) | 6-7 |
| 20 | Assessment of confounding (eg, comparability of cases and controls in studies where appropriate) | 7, Table 3, Table S1 |
| 21 | Assessment of study quality, including blinding of quality assessors, stratification or regression on possible predictors of study results | 6-7, Table S1 |
| 22 | Assessment of heterogeneity | 7, Figures 2-4, Table S1, Figure S1 |
| 23 | Description of statistical methods (eg, complete description of fixed or random effects models, justification of whether the chosen models account for predictors of study results, dose-response models, or cumulative meta-analysis) in sufficient detail to be replicated | 7 |
| 24 | Provision of appropriate tables and graphics | Tables 1-4, Figures 1-4, Table S1, Figure S1 |
| **Reporting of Results** | | |
| 25 | Graphic summarizing individual study estimates and overall estimate | Figures 2-4, Figure S1 |
| 26 | Table giving descriptive information for each study included | Tables 1-2 |
| 27 | Results of sensitivity testing (eg, subgroup analysis) | 11-12 |
| 28 | Indication of statistical uncertainty of findings | 10-12 |
| **Reporting of Discussion** | | |
| 29 | Quantitative assessment of bias (eg, publication bias) | 11, Table S1 |
| 30 | Justification for exclusion (eg, exclusion of non-English language citations) | 5-6, 10 |
| 31 | Assessment of quality of included studies | 11, Table 4 |
| **Reporting of Conclusions** | | |
| 32 | Consideration of alternative explanations for observed results | 13-14 |
| 33 | Generalization of the conclusions (ie, appropriate for the data presented and within the domain of the literature review) | 14-15 |
| 34 | Guidelines for future research | 16 |
| 35 | Disclosure of funding source | 17 |

eAppendix C: Full Search Strategy

**Medline** [1946 – Present]

1. Heart Arrest/

2. Death, Sudden, Cardiac/

3. Out-of-Hospital Cardiac Arrest/

4. (Heart arrest or cardiac arrest or cardiopulmonary arrest or sudden cardiac death).ti,ab.

5. Cardiopulmonary Resuscitation/

6. Heart Massage/

7. Advanced Cardiac Life Support/

8. Defibrillators/

9. Electric Countershock/

10. Resuscitation/

11. (cardiopulmonary resuscitation or cardio pulmonary resuscitation or resuscitation or CPR).ti,ab.

12. Community Participation/

13. Community Health Services/

14. (Communit* or neighborhoo* or neighbourhoo* or natio* or state or states or statewide or county or counties or city or cities or country or regio* or zip code or post code or postal code or municipali* or public health).ti,ab.

15. exp Emergency Responders/

16. (bystander or bystanders or resident or residents or first responder or first responders or lay responder or lay responders).ti,ab.

17. exp Education/

18. (teach* or train* or class or mass gathering or educat* or school or present* or instruct* or skill* or knowledge or audiovisua*).ti,ab.

19. (Health promotion or Mass media or social media or public service announcement* or TV or television or advertisement* or newspaper or radio).ti,ab.

20. (program or programs or service or services or intervention or interventions or initiative or initiatives).ti,ab.

21. 1 or 2 or 3 or 4

22. 5 or 6 or 7 or 8 or 9 or 10 or 11

23. 12 or 13 or 14

24. 15 or 16

25. 17 or 18 or 19 or 20

26. 21 and 22 and 23 and 24 and 25

**Embase** [1966 – Present]

1. heart arrest/ or "out of hospital cardiac arrest"/ or sudden cardiac death/

2. (Heart arrest or cardiac arrest or cardiopulmonary arrest or sudden cardiac death).ti,ab.

3. resuscitation/

4. heart massage/

5. defibrillation/

6. external defibrillator/ or defibrillator/ or automated external defibrillator/

7. (cardiopulmonary resuscitation or cardio pulmonary resuscitation or resuscitation or CPR).ti,ab.

8. community care/ or community program/

9. health care planning/

10. public health/

11. community/

12. (Communit* or neighborhoo* or neighbourhoo* or natio* or state or states or statewide or county or counties or city or cities or country or regio* or zip code or post code or postal code or municipali* or public health).ti,ab.

13. rescue personnel/

14. (bystander or bystanders or resident or residents or first responder or first responders or lay responder or lay responders).ti,ab.

15. exp education/

16. health program/

17. emergency medical services education/

18. (teach* or train* or class or mass gathering or educat* or school or present* or instruct* or skill* or knowledge or audiovisua*).ti,ab.

19. (Health promotion or Mass media or social media or public service announcement* or TV or television or advertisement* or newspaper or radio).ti,ab.

20. (program or programs or service or services or intervention or interventions or initiative or initiatives).ti,ab.

21. 1 or 2

22. 3 or 4 or 5 or 6 or 7

23. 8 or 9 or 10 or 11 or 12

24. 13 or 14

25. 15 or 16 or 17 or 18 or 19 or 20

26. 21 and 22 and 23 and 24 and 25

eAppendix D: Data Extraction Form

| **Study ID:** |  |
| --- | --- |
|  |  |
| **Community Characteristics** | |
| Country |  |
| Geographical location |  |
| Total population size |  |
| Median age of patients with OHCA |  |
| Population density (urban, suburban, rural) |  |
| Socio-economic status of the population |  |
| Other community demographics (i.e., race/ethnicity, tourism, higher rates of co-morbidities, etc.) |  |
| Rates of OHCA per 100 000 |  |
|  |  |
| **Study Characteristics** | |
| Study design (i.e., prospective observational) |  |
| Study start date |  |
| Study end date |  |
|  |  |
| Bystander definition (i.e., laypersons, first responders, EMS) |  |
| OHCA definition – inclusion and exclusion criteria for their analysis |  |
| Data collection source (i.e., registry, EMS records) |  |
| Data collection format/convention (i.e., Utstein) |  |
|  |  |
| Total number of OHCAs in study period |  |
| Number of OHCAs included in study analysis |  |
|  |  |
| Study primary outcomes/aims |  |
| Study secondary outcomes/aims |  |

| **Community-based Interventions** | |
| --- | --- |
| First intervention start date |  |
| Last intervention end date (or study end date if ongoing) |  |
|  |  |
| **Intervention – CPR Training Initiative** | **Y/N** |
| Timeline |  |
| Description of intervention (who, what, where) |  |
| Number of people trained |  |
|  |  |
| **Intervention – AED Training Initiative** | **Y/N** |
| Timeline |  |
| Description of intervention (who, what, where) |  |
| Number of people trained |  |
|  |  |
| **Intervention – School-based Training Initiative** | **Y/N** |
| Timeline |  |
| Description of intervention (who, what, where) |  |
| Number of people trained |  |
|  |  |
| **Intervention – PAD program** | **Y/N** |
| Timeline |  |
| Description of intervention (who, what, where) |  |
| Number of AEDs deployed |  |
|  |  |
| **Intervention – Notification system or application for nearby OHCAs** | **Y/N** |
| Timeline |  |
| Description of intervention (who, what, where) |  |
| Number of people signed up |  |
|  |  |
| **Intervention – DA-CPR** | **Y/N** |
| Timeline |  |
| Description of intervention (who, what, where) |  |
|  |  |
| **Intervention – Mass Media or Awareness Campaign** | **Y/N** |
| Timeline |  |
| Description of intervention (who, what, where) |  |
|  |  |
| **Intervention – Mass Training Event** | **Y/N** |
| Timeline |  |
| Description of intervention (who, what, where) |  |
| Number of people trained |  |
|  |  |
| **Intervention – Legislative changes mandating CPR or AED training** | **Y/N** |
| Timeline |  |
| Description of intervention (who, what, where) |  |
| Number of people trained |  |
|  |  |
| **Health System Interventions** | |
| **Intervention – EMS or first responder interventions** | **Y/N** |
| Timeline |  |
| Description of intervention (who, what, where) |  |
|  |  |
| **Intervention – Hospital-based interventions** | **Y/N** |
| Timeline |  |
| Description of intervention (who, what, where) |  |
|  |  |
| **Outcomes relevant to the review** | |
| **Bystander CPR rates** | **Y/N** |
| Before rates |  |
| After rates |  |
| P value |  |
| Comments (dates collected, OHCA characteristics, etc.) |  |
|  |  |
| **Bystander AED rates** | **Y/N** |
| Before rates |  |
| After rates |  |
| P value |  |
| Comments (dates collected, OHCA characteristics, etc.) |  |
|  |  |
| **Survival rates (to hospital discharge)** | **Y/N** |
| Before rates |  |
| After rates |  |
| P value |  |
| Comments (dates collected, OHCA characteristics, etc.) |  |
|  |  |
| **Survival rates (to 30 days)** | **Y/N** |
| Before rates |  |
| After rates |  |
| P value |  |
| Comments (dates collected, OHCA characteristics, etc.) |  |
|  |  |
| **Survival with a favorable neurological outcome** |  |
| Before rates |  |
| After rates |  |
| P value |  |
| Comments (dates collected, OHCA characteristics, etc.) |  |
|  |  |
| **Other** | |
| Other reported outcomes and their values |  |
|  |  |
| Author explanation of outcomes (if note-worthy) |  |
|  |  |
| Key conclusions of the authors |  |
|  |  |
| Other comments |  |

eAppendix E: Quality Assessment Criteria

**Newcastle-Ottawa Quality Assessment Scale**

Note: A study can be awarded a maximum of one star for each numbered item within each category.

**Selection**

1. Representativeness of the exposed cohort
2. truly representative of the average layperson in the community (NOT only first responders, EMS, healthcare providers, students, etc.) ★
3. somewhat representative of the average layperson in the community ★
4. selected group of users (only first responders, EMS, healthcare providers, students, etc.)
5. no description of the derivation of the cohort
6. Selection of the non exposed cohort
7. drawn from the same community as the exposed cohort (a before-and-after comparison) ★
8. drawn from a different source (i.e., a neighbouring community)
9. no description of the derivation of the non exposed cohort
10. Ascertainment of exposure
11. secure record: regional cardiac arrest registry, EMS records ★
12. structured interview ★
13. written self report
14. no description
15. Demonstration that outcome of interest was not present at start of study
16. yes ★
17. no

**Comparability**

1. Comparability of cohorts on the basis of the design or analysis
2. Multivariate analysis of average age and sex of OHCA patients; finds that there is no significant difference between pre-and post-cohorts or evaluates their effects as non-significant ★

**Outcome**

1. Assessment of outcome
2. independent blind assessment ★
3. record linkage: regional cardiac arrest registry, EMS records ★
4. self report
5. no description
6. Was follow-up long enough for outcomes to occur
7. yes: greater than six months ★
8. no
9. Adequacy of follow up of cohorts
10. population unchanged over study period ★
11. little variation in proportion and size of population reported: minor annual population variation ★
12. change in the catchment area or source of data (registry/EMS records) used
13. no statement regarding population stability

eAppendix F: GRADE Evidence to Decision Framework

| **Question** | |
| --- | --- |
| **Should community-based intervention with or without health system interventions be used for enhancing outcomes following out-of-hospital cardiac arrest?** | |
| **Population:** | Individuals experiencing out-of-hospital cardiac arrest within a defined community (town, county, state, or country) |
| **Intervention:** | Community-based interventions with or without health system interventions |
| **Comparison:** | Prior to or without community-based intervention(s) |
| **Main outcomes:** | Bystander-CPR rates (critical); survival rates (critical); survival with favorable neurological outcome rates (critical); bystander-AED rates (important) |
| **Setting:** | Out-of-hospital |
| **Perspective:** | Healthcare system |
| **Conflict of interests:** | None declared |

**Assessment**

| **Problem**  Is the problem a priority? | |
| --- | --- |
| **Judgement** | **Research evidence** |
| ○ No ○ Probably no ○ Probably yes ● Yes ○ Varies ○ Don't know | Out-of-hospital cardiac arrest (OHCA) is a leading cause of morbidity and mortality.^1,51^ OHCA impacts nearly 400 000 Americans annually; worldwide rates of OHCA vary significantly, effecting 30 to 97 individuals per 100 000 person-years.^3^ Survival remains low at approximately 10%^4^. Bystander-CPR and bystander-AED are known to enhance outcomes following OHCA.^11^ Many organizations, communities, and entire countries have identified this area for improvement and have taken steps to enhance OHCA outcomes via community- and systems-based interventions. |
| **Desirable Effects**  How substantial are the desirable anticipated effects? | |
| **Judgement** | **Research evidence** |
| ○ Trivial ○ Small ● Moderate ○ Large ○ Varies ○ Don't know | Community- and systems-based interventions are associated with improvements in survival and survival with favorable neurological outcome; these findings are noted consistently though absolute effect size varies. |
| **Undesirable Effects**  How substantial are the undesirable anticipated effects? | |
| **Judgement** | **Research evidence** |
| ○ Large ○ Moderate ● Small ○ Trivial ○ Varies ○ Don't know | No undesirable effects related to such interventions were noted in the literature. The authors have hypothesized some potential undesirable effects though they are currently unsubstantiated to our knowledge. One, it is possible that performing bystander-CPR could cause physical trauma to the ribs of the patient. Two, with more survivors, there could also be a greater number of individuals with neurological or other deficits following OHCA. Finally, it is possible that costs associated with these interventions may reduce funding available for other healthcare programs or services. |
| **Certainty of evidence**  What is the overall certainty of the evidence of effects? | |
| **Judgement** | **Research evidence** |
| ● Very low ○ Low ○ Moderate ○ High ○ No included studies | The certainty of evidence among outcomes was evaluated as low or very low. Evidence certainty was downgraded primarily due to the observational nature of included studies, significant heterogeneity between studies, and risk of bias. However, there was a consistent, positive direction of effect and dose-response relationship observed among outcomes or interest. The full assessment is available in Table S1. |
| **Values**  Is there important uncertainty about or variability in how much people value the main outcomes? | |
| **Judgement** | **Research evidence** |
| ○ Important uncertainty or variability ○ Possibly important uncertainty or variability ○ Probably no important uncertainty or variability ● No important uncertainty or variability | Patients and their families desire enhanced OHCA outcomes in terms of survival and quality of life (which can be impacted if there are neurological or other sequelae post-OHCA).^52^ A 2021 study surveyed OHCA survivors, their families, and healthcare providers and found that the top 2 research priorities were to identify the most effective mechanisms for improving bystander-CPR and which interventions improve resuscitation and survival outcomes.^52^ |
| **Balance of effects**  Does the balance between desirable and undesirable effects favor the intervention or the comparison? | |
| **Judgement** | **Research evidence** |
| ○ Favors the comparison ○ Probably favors the comparison ○ Does not favor either the intervention or the comparison ● Probably favors the intervention ○ Favors the intervention ○ Varies ○ Don't know | The data shows a clear positive trend between the implementation of community- and systems-based interventions and enhanced rates of bystander-CPR and bystander-AED use as well as clinical outcomes such as survival and survival with favorable neurological outcome. As mentioned, there is currently limited evidence of undesirable effects associated with such interventions. |
| **Resources required**  How large are the resource requirements (costs)? | |
| **Judgement** | **Research evidence** |
| ○ Large costs ○ Moderate costs ○ Negligible costs and savings ○ Moderate savings ○ Large savings ○ Varies ● Don't know | At this time, we are unable to accurately evaluate the costs and resources required for community- and systems-based interventions. There have been few cost analyses completed on such initiatives and they reported a wide variation in cost. Two estimates of the cost per quality adjusted life-years (QALYs) for community-based interventions ranged from $20 000^53^ and $200 000.^54^ Further, as the combination of interventions as well as how they are implemented varies across initiatives, the resources required will be unique for each.  As such, resources utilization has not been considered within our assessment; we recommend that resource allocation be considered at the level of the decision-making entities. |
| **Certainty of evidence of required resources**  What is the certainty of the evidence of resource requirements (costs)? | |
| **Judgement** | **Research evidence** |
| ○ Very low ○ Low ○ Moderate ○ High ● No included studies | As mentioned above, resources utilization has not been considered within our assessment; we recommend that resource allocation be considered at the level of the decision-making entities. |
| **Cost effectiveness**  Does the cost-effectiveness of the intervention favor the intervention or the comparison? | |
| **Judgement** | **Research evidence** |
| ○ Favors the comparison ○ Probably favors the comparison ○ Does not favor either the intervention or the comparison ○ Probably favors the intervention ○ Favors the intervention ○ Varies ● No included studies | As mentioned above, resources utilization has not been considered within our assessment; we recommend that resource allocation be considered at the level of the decision-making entities. |
| **Equity**  What would be the impact on health equity? | |
| **Judgement** | **Research evidence** |
| ○ Reduced ○ Probably reduced ○ Probably no impact ● Probably increased ○ Increased ○ Varies ○ Don't know | Studies have noted higher rates of out-of-hospital cardiac arrest and lower rates of bystander-CPR areas with lower socioeconomic status as well as among racial minorities.^34,49^ There is evidence to suggest that community- and systems-based interventions may increase rates of bystander-CPR more in such areas than in the general population, thereby reducing these health inequities.^49^ |
| **Acceptability**  Is the intervention acceptable to key stakeholders? | |
| **Judgement** | **Research evidence** |
| ○ No ○ Probably no ● Probably yes ○ Yes ○ Varies ○ Don't know | As these interventions are system-wide, there are numerous stakeholders to consider: community members, schools, local policy makers, emergency medical services, first responders (fire and police), and various levels of hospital staff. Improving OHCA outcomes has been reported as a priority among community members and healthcare providers.^52^ However, there is a possibility that implementing new policies and programs may meet resistance among staff opposed to training or additional responsibilities. There could also be concern regarding start-up costs among local leaders and politicians. We hypothesize that the predicted benefit (improved OHCA outcomes) would provide greater motivation to key stakeholders than the potential initial barriers. |
| **Feasibility**  Is the intervention feasible to implement? | |
| **Judgement** | **Research evidence** |
| ○ No ○ Probably no ● Probably yes ○ Yes ○ Varies ○ Don't know | Numerous interventions have been ongoing for over a decade without interruption.^13,14,41,44^ The interventions typically are planned and executed by local leaders and take the unique strengths and challenges of a community into account. As such, community-wide, systems-based interventions are a feasible and sustainable approach to improving OHCA outcomes. |

**Type of recommendation**

| Strong recommendation against the intervention | Conditional recommendation against the intervention | Conditional recommendation for either the intervention or the comparison | Conditional recommendation for the intervention | **Strong recommendation for the intervention** |
| --- | --- | --- | --- | --- |
| ○ | ○ | ○ | ○ | **●** |

**Conclusions**

| **Recommendation** |
| --- |
| We strongly recommend that communities, counties, states, and countries assess their current pre-hospital strategy for OHCA care and consider implementing community-based interventions, especially when combined with health system interventions, to improve outcomes following OHCA. Given the success of a wide variety and combination of community-based interventions reported in included studies, interventions can be individualized to meet the unique needs of a community given their resources. We encourage that interventions are sustainable and continued long-term for greatest impact. |
|  |
| **Justification** |
| On evaluation, community- and systems-based interventions are associated with improved OHCA outcomes. Though effect size cannot be determined with certainty due to the significant heterogeneity between studies, the direction of effect was consistently positive. Further, undesirable effects were not described in the reviewed literature and theorized consequences are minimal when compared with the potential benefits (survival and favorable neurological outcome). Community- and systems-based interventions seem to be a feasible option to improve OHCA morbidity and mortality. |

| **Subgroup considerations** |
| --- |
| Not applicable. |
| **Implementation considerations** |
| Initiatives should try to incorporate both community- and systems-based interventions and aim for ongoing, long-term programs; studies with these characteristics were associated with greater increases among outcomes. |

| **Monitoring and evaluation** |
| --- |
| Not applicable. |
| **Research priorities** |
| Further research is required to elucidate which specific interventions are associated with the greatest improvement in OHCA outcomes. A cost-analysis of the various interventions is also needed. This knowledge will be important to inform future initiatives to maximize impact given the limited funding and resources in healthcare systems. |

Supplemental Table 1. Evidence Profiles for Outcomes.

| **Certainty assessment** | | | | | | | | **Summary of findings** | | | | |
| --- | --- | --- | --- | --- | --- | --- | --- | --- | --- | --- | --- | --- |
| **Participants  (studies)** | **Risk of bias** | **Inconsistency** | **Indirectness** | **Imprecision** | **Other considerations** | **Overall certainty of evidence** | **Study event rates (%)** | | | **Relative effect (95% CI)** | **Anticipated absolute effects** | |
|  |  |  |  |  |  |  | **Prior to CBIs or HSIs** | | **After CBIs or HSIs** |  | **Risk prior to CBIs or HSIs** | **Risk difference after CBIs or HSIs** |
| **Bystander-CPR rates** (critical outcome) | | | | | | | | | | | | |
| 285 752  (14 observational studies) | serious ^*^ | serious ^†^ | not serious | not serious | strong association  dose response gradient | ⨁⨁◯◯ LOW | 42 011/ 123 954 (33.9%) | | 77 860/ 161 798 (48.1%) | **OR 2.26** (1.74 to 2.94) | 339 per 1000 | **198 more per 1000** (from 133 to 262 more) |
| **Survival rates** (critical outcome) | | | | | | | | | | | | |
| 79 206 (10 observational studies) | serious ^*^ | serious ^†^ | not serious | not serious | dose response gradient | ⨁◯◯◯ VERY LOW | 2919/  32 294 (9.0%) | | 4619/  46 912 (9.8%) | **OR 1.59** (1.20 to 2.10) | 90 per 1000 | **46 more per 1000** (from 16 to 82 more) |
| **Survival with favorable neurological outcome rates** (critical outcome) | | | | | | | | | | | | |
| 272 882 (8 observational studies) | serious ^*^ | serious ^†^ | not serious | not serious | dose response gradient | ⨁◯◯◯ VERY LOW | 2639/  117 317 (2.2%) | | 5193/ 155 565 (3.3%) | **OR 1.42** (1.06 to 1.90) | 22 per 1000 | **9 more per 1000** (from 1 to 19 more) |
| **Bystander-AED rates** (important outcome) | | | | | | | | | | | | |
| 37 882 (5 observational studies) | serious ^*^ | not serious | not serious | not serious | none | ⨁◯◯◯ VERY LOW | 259/  12 393 (2.1%) | | 1107/  25 489 (4.3%) | **OR 2.08** (1.44 to 3.01) | 21 per 1000 | **22 more per 1000** (from 9 to 39 more) |

CBIs = community-based interventions; CI = confidence interval; HSIs = health system interventions OR = odds ratio

***Explanations***

* Failure to adequately control or adjust for confounding factors due to study design.

† Considerable heterogeneity present (defined as I-squared 75-100%); some but not all heterogeneity explained with sub-group analysis.


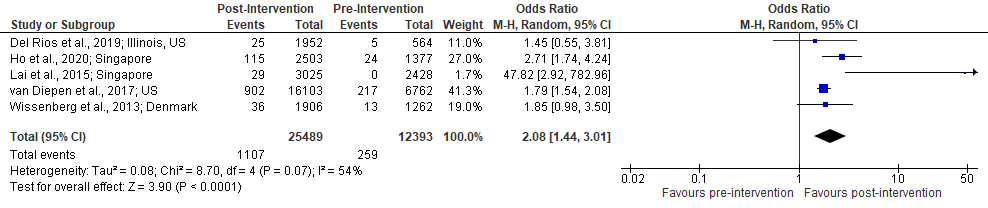


Supplemental Figure 1. Forest plot of studies reporting Bystander-AED rates
